# Detection of Water Changes in Plant Stems In Situ by the Primary Echo of Ultrasound RF with an Improved AIC Algorithm

**DOI:** 10.3390/s23010020

**Published:** 2022-12-20

**Authors:** Danju Lv, Jiali Zi, Mingyuan Gao, Rui Xi, Xin Huang

**Affiliations:** College of Big Data and Intelligent Engineering, Southwest Forestry University, Kunming 650224, China

**Keywords:** non-destructive detection, RFID, AIC, ultrasonic, water changes in plant stem

## Abstract

The detection of water changes in plant stems by non-destructive online methods has become a hot spot in studying the physiological activity of plant water. In this paper, the ultrasonic radio-frequency echo (RFID) technique was used to detect water changes in stems. An algorithm (improved hybrid differential Akaike’s Information Criterion (AIC)) was proposed to automatically compute the position of the primary ultrasonic echo of stems, which is the key parameter of water changes in stems. This method overcame the inaccurate location of the primary echo, which was caused by the anisotropic ultrasound propagation and heterogeneous stems. First of all, the improved algorithm was analyzed and its accuracy was verified by a set of simulated signals. Then, a set of cutting samples from stems were taken for ultrasonic detection in the process of water absorption. The correlation between the moisture content of stems and ultrasonic velocities was computed with the algorithm. It was found that the average correlation coefficient of the two parameters reached about 0.98. Finally, living sunflowers with different soil moistures were subjected to ultrasonic detection from 9:00 to 18:00 in situ. The results showed that the soil moisture and the primary ultrasonic echo position had a positive correlation, especially from 12:00 to 18:00; the average coefficient was 0.92. Meanwhile, our results showed that the ultrasonic detection of sunflower stems with different soil moistures was significantly distinct. Therefore, the improved AIC algorithm provided a method to effectively compute the primary echo position of limbs to help detect water changes in stems in situ.

## 1. Introduction

The water content of plant stems is a major index of the physiological activity of plant water, especially in precision agriculture [1,2,3]. Methods for detecting stem water content include drying [4], gamma rays [5], nuclear magnetic resonance (NMR) [6,7,8], X-ray computed tomography [9], resistance [10], time-domain reflectometry (TDR) [11], frequency-domain (FD) capacitance [12,13,14] and infrared detection [15]. Of the above methods, drying constitutes in vitro detection, which cannot detect the dynamic changes in the stem water content. For online detection, the time-domain reflection method and frequency capacitance method could track the changes in stem water content. However, the probes in both methods need to be inserted into the plant stem, which could affect plants’ physiological activity [16,17], resulting in detection errors in dynamic changes [18]. It was pointed out that the consistency of the detection of stem water content was influenced by the insertion depth of the probe and the structure of the plant stem [17]. The gamma-ray method and NMR method, two destructive detection methods, had high accuracy but were expensive and difficult to utilize for long-term detection. The infrared method could detect the liquid water and ice content of plant stems in situ, in real time with micro-destruction [15]. Recently, non-destructive online detection methods were explored based on the principle of the standing wave rate and frequency-domain capacitance. Both groups of researchers pointed out that both methods of tracking water changes in stems are affected by differences in stem structure [19,20].

The change in stem water content was closely related not only to the physiological and external conditions of the plant, but also to the morphological and structural characteristics of the plant stems [21,22]. Ultrasonic detection was characterized by non-destructive online detection, which was widely used to detect the internal structure and properties of the material. The advantage of ultrasonic detection met the detection needs by tracking the water changes and structure of stems. Researchers have tried to apply it to detect the features of stems. Sandoz measured wood’s strength performance through properties such as parallel grain, the flexural modulus of elasticity and bending strength by measuring ultrasonic velocity with a low-frequency wave in wood in a longitudinal direction [23]. Researchers found that the ultrasonic velocity decreased with the increase in wood water content [22,24]. They also found that the ultrasonic speed and attenuation are closely related to the detection direction of wood. The ultrasonic wave has the smallest attenuation and highest velocity in the axial wood detection; in the radial direction, the attenuation is the largest, and the velocity is the lowest [25,26]. The latest research has pointed out that the characteristics of the ultrasonic attenuation of wood are closely related to the structure of plant stem bodies [27]. In 2014, Guillaume first studied the changes in plant stems by ultrasonic detection under freezing conditions. The study detected the process of freezing and thawing of cut stems by ultrasonic velocity and attenuation. The authors of this study detected the regulation of stems with the ultrasonic detection technique. In 2021, it was found that ultrasound is a reliable non-destructive method commonly used to evaluate the state of a piece of wood. The effect of the moisture content (MC) on the timber wave velocity, which is different depending on the wood species, had been defined by a model [15,22].

Ultrasound provided an effective means for non-destructive dynamic detection [28,29,30,31] for dynamically tracking water changes in stems [7,8,32]. However, due to stems being non-metal materials with anisotropic heterogeneous features, there were widely different and complicated acoustic impedance interfaces [33]. The interfaces lead to multiple refractions and reflections when the ultrasonic wave spreads in plant stems [9,10]. Therefore, the complex propagation in stems made it challenging to extract the location of the primary ultrasonic echo [11,12,13]. At the same time, it was the most basic ultrasonic parameter for the water changes in stems. At present, ultrasonic primary echo detection methods are usually divided into two kinds: the envelope method [16,17] and the Akaike information (Akaike) method [18,19]. The envelope method was based on the threshold level to determine the location of the primary echo [20]. However, it was not convenient to use the envelope method because the wide variations in stems in different plants [34,35] led to various thresholds, which should be calibrated before detection in other plant stems [20]. In contrast, the AIC method does not require the threshold to be set. It uses the global minimum to distinguish the noise and ultrasonic echo effectively and then locate the position of the ultrasonic echo [6,36,37].

For different detecting conditions, many improved algorithms of the AIC method were proposed. In this paper, we adopted the ultrasonic reflection method instead of transmission to avoid the requirement of probe alignment. Reflection detection would make it more difficult to detect the position of a primary echo than transmission because the propagation path of reflection was longer than that of communication, which caused the weak ultrasound to be embedded in solid interferences. Therefore, we proposed an improved hybrid differential AIC algorithm to locate the ultrasonic primary echo position in stems using reflection detection. The ultrasonic velocity was calculated based on the primary echo’s location. After that, we applied the improved AIC to obtain the site of the primary echo of a woodblock and analyzed the correlation between the changes in water content and ultrasonic velocity. Finally, the ultrasonic primary echo position was measured in sunflowers’ stems to track daytime water changes.

## 2. Materials and Methods

### 2.1. Ultrasonic Detection Principle

The prosperity of the ultrasonic wave is different in various materials. The stem can be studied as a mixture of wood fiber, liquid and ice [38]. The water regulation process of stems is the change in the above mixture components in proportion. The difference between water deficit and filling in stems can be detected using ultrasonic waves [4,29,39]. When the ultrasonic signal propagation forms different ultrasound interface reflection echoes in additional water content, the primary echo positions in stems change. In this paper, the change in water content was studied by determining the position of the ultrasonic primary echo signal of stems.

### 2.2. Ultrasonic Detection of Water Content Changes in Stems

Ultrasonic wave propagation in a solid medium is determined by the density and elastic properties of the medium. Ultrasonic velocity in a solid medium is

(1) $$v_{l}=\sqrt{\frac{E}{\rho }}\sqrt{\frac{1-\sigma }{\left(1+\sigma \right)\left(1-2\sigma \right)}}$$

where $v_{l}$ is the propagation velocity of the ultrasonic wave in the longitudinal direction, *E* is the modulus of elasticity, *ρ* is the density of the medium and *σ* is Poisson’s ratio.

We hypothesized that when the water content of stems changes, Poisson’s ratio and the modulus of elasticity *E* of the same stem are regarded as constants [4,39]. Therefore, the water content dominates ultrasonic velocity because the water content changes the medium’s density *ρ* [28,29] for the detected stems.

According to Formula (1), the increasing density eventually leads to decreased ultrasonic velocity in stems [39]. On the contrary, the velocity rises when water is deficient, causing the viscosity to fall. Therefore, the water regulation of the plant stem can be obtained by detecting the ultrasonic speed in the plant stem.

In detection, the time of the ultrasonic primary echo position and the distance of the ultrasonic wave propagation are used calculate the ultrasonic velocity:

(2) $$v_{l}=\frac{2D}{t}$$

where 2D is the linear distance of the ultrasonic echo in the medium and t is the ultrasonic primary echo position time.

### 2.3. Detection of the Position of Ultrasonic Primary Echo for Stems Based on AIC Algorithm

Because the stem is a non-metallic, heterogeneous, anisotropic material, the path of ultrasonic echo propagation within it is more complex than that in metallic, homogeneous and isotropic materials. Similarly, the energy attenuation of stems is more serious. Therefore, the path and attenuation make it challenging to distinguish the primary echo wave from other multiple ultrasonic echo signals [13,18], affecting the detection of the time of the primary echo.

#### 2.3.1. Detection of Ultrasonic Echo Time Based on AIC

In AIC, the echo signal is assumed to be the local autocorrelation stationary signal alternated with the noise. The AIC for the timely detection of the primary ultrasonic echo is represented as a function of a merging point k.

(3) $$AIC\left(k\right)=k\cdot log\left(var\left(s\left(1,k\right)\right)\right)+\left(N-k+1\right)\cdot \log\left(var\left(s\left(k+1,N\right)\right)\right)$$

where $s\left(1,k\right)$ represents the samples of the ultrasonic echo from the 1st sample to the k-th sample, N is the length of the samples of the ultrasonic echo, and k is the sampling point sequence number from 1 to N. In the function, the following formula expresses the var.

(4) $$var\left(s\left(i,j\right)\right)=\sigma_{j-1}^{2}=1/(j-i)\overset{j}{\underset{l=i}{\sum }}{\left(s\left(l,l\right)-\bar{s}\right)}^{2}$$

where $\bar{s}$ is the mean value of $s\left(i,j\right)$, i≤j,i=1,…,N,j=1,…,N.

The AIC(k) of stems is calculated using Formula (3) on the ultrasonic echo sampling signal $s\left(l,k\right)$. The global minimum AIC(k) is the time of the primary echo. This is when the ultrasonic wave injuncts with the noise. After determining the time of the primary echo, we can calculate the ultrasonic velocity and other parameters of ultrasound echo in the stem, laying the foundation for the effective and accurate construction of ultrasound images of plant stems.

#### 2.3.2. Ultrasonic Detection of Plant Stems Based on AIC Algorithm

**Figure 1:**
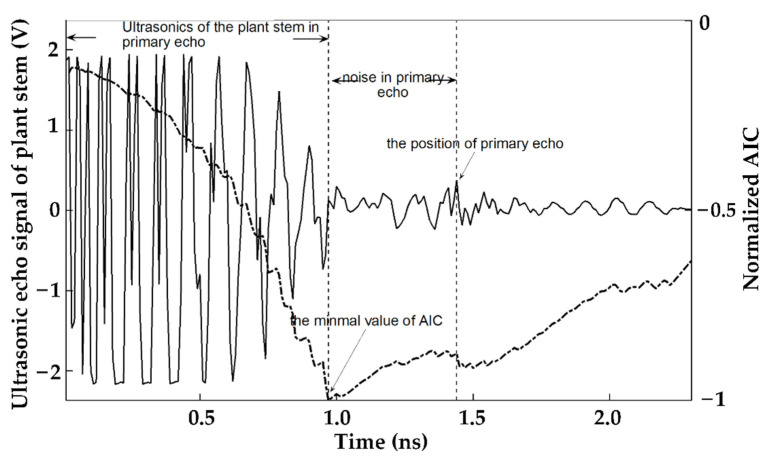
Ultrasound in 2 cm block and the AIC curve.

The ultrasonic signal of the plant stem is presented in Figure 1. It shows that the ultrasonic wave and the noise alternately appear. The noise, due to the strong attenuation of the plant stem, is the ultrasonic wave drowned by noise. In Figure 1, the position determined by the minimal AIC is not the primary ultrasonic echolocation but the junction of the ultrasound and noise. The position of the primary natural echo is found at the first inflection point convex after the position of the minimal of AIC. Therefore, the classical AIC algorithm cannot accurately detect the position of the ultrasonic echo of the plant stem.

### 2.4. Hybrid Difference AIC Algorithm

The hybrid difference AIC algorithm highlights the position of the primary echo by the difference mixing with the AIC. The hybrid model used to pick the location of the primary echo comprises the following sequences:

1. Calculate AIC(n) values (AIC(n), n = 1, …, N);
2. Extract AIC(n) from the sample of the minimal value (AICmin(index)) to the last sample N to form the *AICseg*(*i*), i = index, …, N;
3. Compute the M order differences of *AICseg*(*i*), to obtain *Diff_AICseg_*(*i*);
  (5) $$Diff_{AICseg}\left(i\right)=△M\left(i\right)={∆}^{M-1}AICseg\left(i+1\right)-{∆}^{M-1}AICseg\left(i\right)$$
  where △M is the M order difference.
4. Compute the envelope of *Diff_AICseg_*(*i*) and normalize it;
  (6) $$Envelope\left(i\right)={\left(Diff_{AICseg}\left(i\right)\right)}^{3}$$
  Because of the phase change of the ultrasonic echo signal, the upper and lower envelopes are retained.
5. Multiply *Envelope*(*i*) and *AICseg*(*i*),
  (7) $$Mixed_{AIC}\left(i\right)=Envelope\left(i\right)\times AIC_{seg}\left(i\right)$$
6. Pick the maximum of the *Mixed_AIC_*(*i*). The time of the maximum is the time of primary echo.

### 2.5. Ultrasonic Detection System

**Figure 2:**
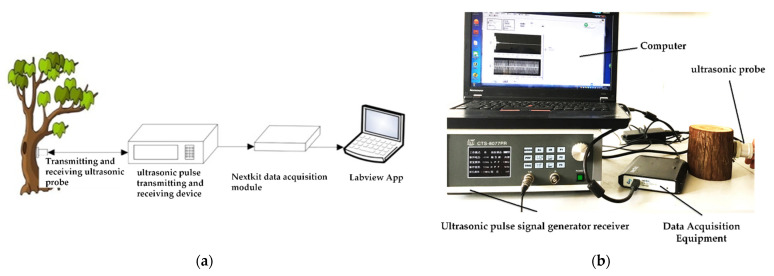
Ultrasonic stem water content detection system. (**a**) Flow chart; (**b**) equipment drawing.

**Figure 3:**
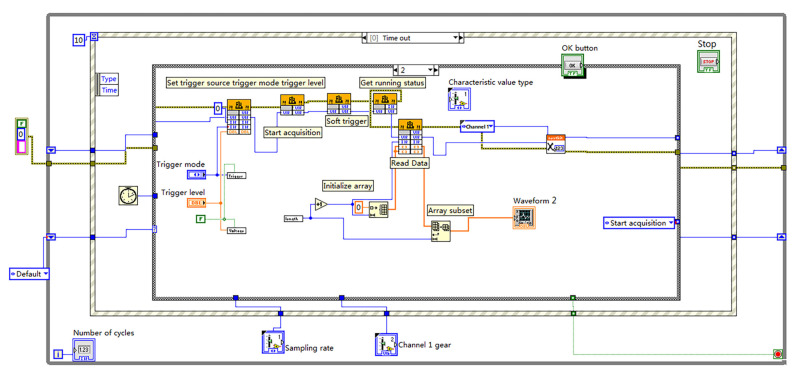
Ultrasonic stem water content detection system on Labview.

An ultrasound apparatus for detecting the water content of stems was designed, as shown in Figure 2 [5]. It consists of a non-metallic ultrasonic probe of 1 MHz, an ultrasonic pulse transmitting and receiving device (CTS-8077PR), a data acquisition module, and a PC machine. The sampling depth of the detecting system was set to 2000 samples per ultrasonic pulse. The sampling frequency was 10 MHz. The system stores and analyzes the ultrasonic echo signal, as shown in Figure 3.

## 3. Results

### 3.1. Simulation Experiment

Firstly, we evaluated the improved AIC algorithm by applying it to detect the time of primary echo of nine sets of simulated ultrasonic signals in MATLAB 2020a. 

Secondly, we applied the algorithm to track the changes in water content in the samples of the stems in the water immersion experiment. Two sets of samples were taken. The first sets are three samples of 2 cm × 4 cm × 10 cm stems with different densities. The thicknesses of ultrasonic detection were all 2 cm. The experiment compared the time of the primary echo and the ultrasonic velocity before and after 24 h immersion. The second sets of samples were cylindrical samples cut from stems. Their sizes were 6 cm × 6 cm, 7 cm × 7 cm and 10 cm × 10 cm. Because the water content changed significantly in the initial immersion, the detection time interval was set to 10 or 20 min and detected six times; in the middle stage of immersion, the detection interval was 3~4 h, detected two times; and the final detection time interval was 24 h. The experiment stopped when the increased amount of water was less than 10 g for 24 h.

Finally, the algorithm was applied to detect the water content changes in stems of living sunflowers in situ with different soil moistures. Two healthy pot-planted sunflowers were selected as samples. The day before the detection, one of the samples was usually watered with 100 mL of water, and the other was not watered. The instrument for measuring soil moisture is ZY-05, and the mechanism for detecting air temperature and humidity is AR837. The ultrasonic probe was placed 6 cm along the stems from the soil’s surface. The circumferences of the samples were 4 cm and 6 cm. The detection period was from 9:00 to 19:00.

### 3.2. Ultrasonic Detection for Samples of Plant Stem

**Figure 4:**
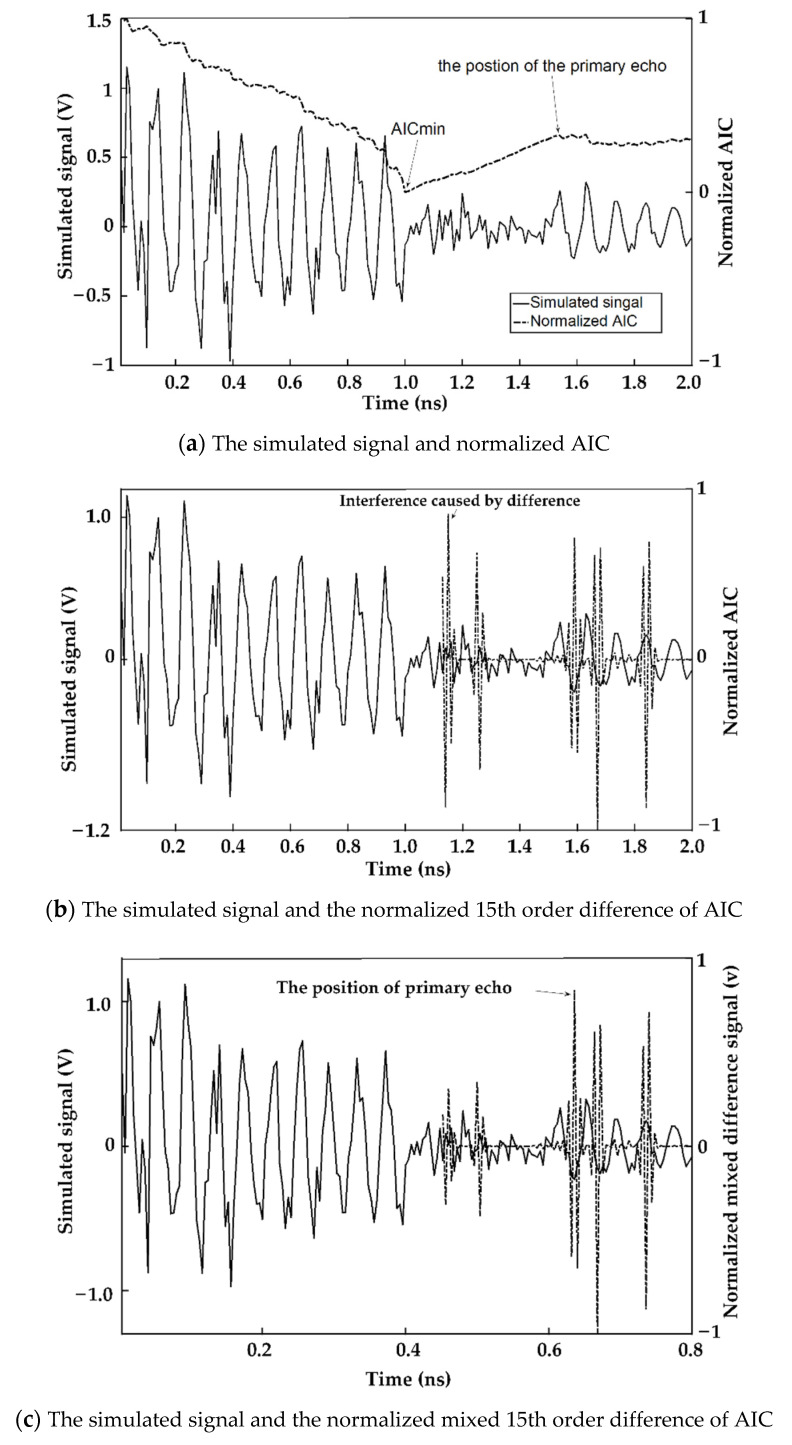
Process of mixed difference AIC. (**a**) shows one of the simulated waves, where 0 ns to 1 ns shows the ultrasound in the plant stem; 1.01 ns to 1.52 ns shows simulated the ultrasound drowned by noise; the 1.53~2.0 ns signal simulates the primary echo signal. The value of 1.53 ns is the ultrasonic primary echo position. (**b**) The AIC curve took the 4th order difference. (**c**) The mixed AIC curve was obtained from *Diff_AICseg_* multiplying AIC.

Figure 4b shows the time when the maximum appeared, as found using *Diff_AICseg_*. However, it was also found that some interference near 1.10 ns and 1.25 ns of *Diff_AICseg_* occurred after the difference operation. Figure 4c shows that the hybrid differential AIC signal, named *Mixed_AIC_*, can effectively suppress the above interference. The simulated experimental results in Table 1 show that the average error of the primary echo position calculated by the algorithm is 0.1 ns. The hybrid difference AIC can effectively and accurately obtain the primary echo position of the signal.

**Figure 5:**
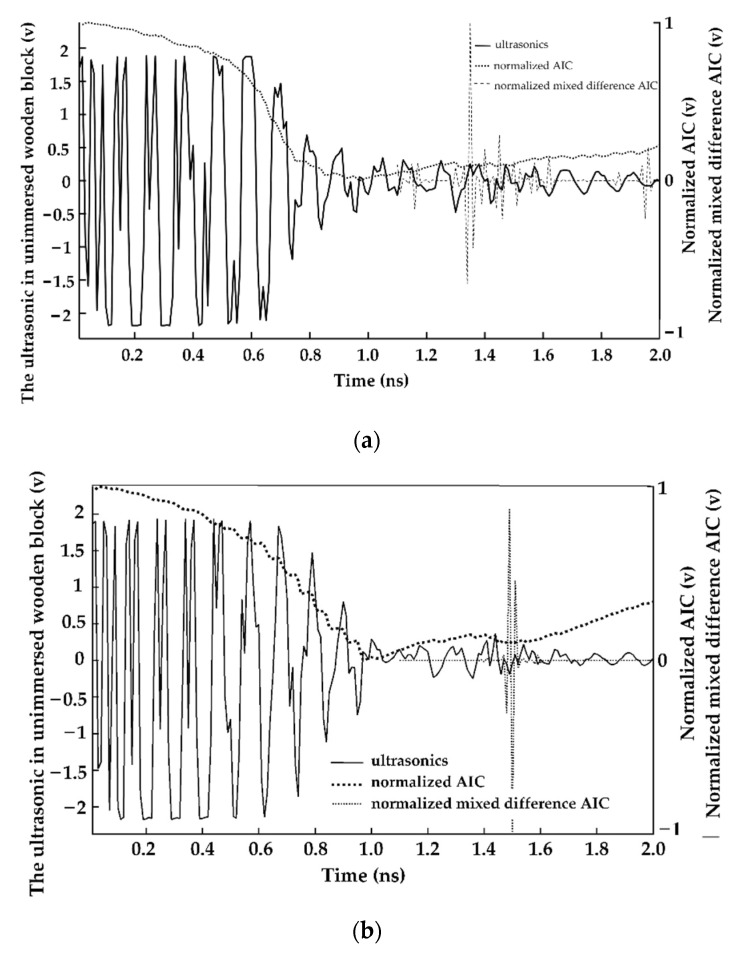
Ultrasounds and curves of AIC and mixed difference AIC of wood block. (**a**) Non-immersed wooden block; (**b**) wooden block immersed for 24 h in water.

Figure 5 shows that the hybrid difference AIC algorithm effectively suppresses the interference of the differential AIC. It is easy to detect the position of the primary echo by picking the maximum of the *Mixed_AIC_*. The time of the primary echo is 1.31 ns, compared to 1.46 ns after 24 h of immersion.

Table 2 shows that the water absorption of truncated stems in the first 1 h after immersing was obvious, especially for the 6 cm diameter of the sample in the first 20 min. The mass increased by nearly 14 g, and the ultrasonic velocity decreased from 4706 m/s to 3859 m/s. The volume of water content increased from zero to 8%. With the increase in soaking time, the water absorption capacity decreases gradually. After 9 days of soaking, the mass of the 6 cm diameter of the sample increases by only 3.8 g, and the final ultrasonic velocity was 1500 m/s. The volume water content of the sample was 42%.

Calculation formula for volume water content:

(8) $$\theta =(m_{A}-m_{B})/\left(\beta V\right)$$

where $m_{A}$ is the mass of the sample after soaking, and $m_{B}$ is the mass of the sample before soaking. *β* is the density of the water, and *V* is the volume of the sample.

**Figure 6:**
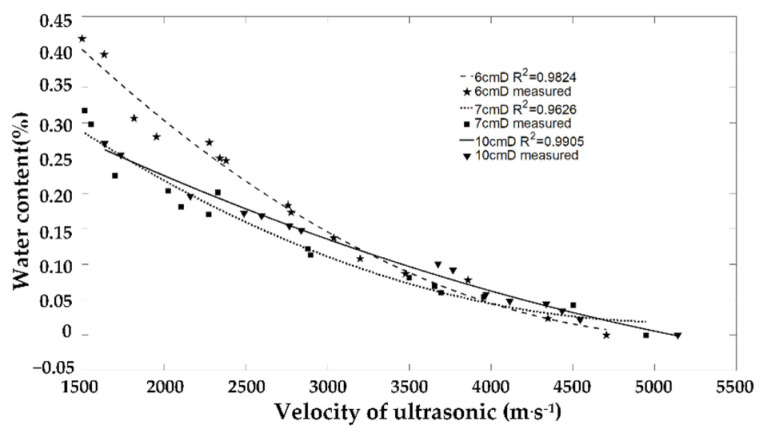
Relationship between plant stem water content and ultrasonic velocity.

Figure 6 shows that the variation of ultrasonic echo velocity and stem water content of stem samples during water absorption can be effectively fitted by two polynomials. The average correlation coefficient reached about 0.98.

### 3.3. Location Detection of Ultrasonic Echo from the Stem Body of a Living Sunflower

**Figure 7:**
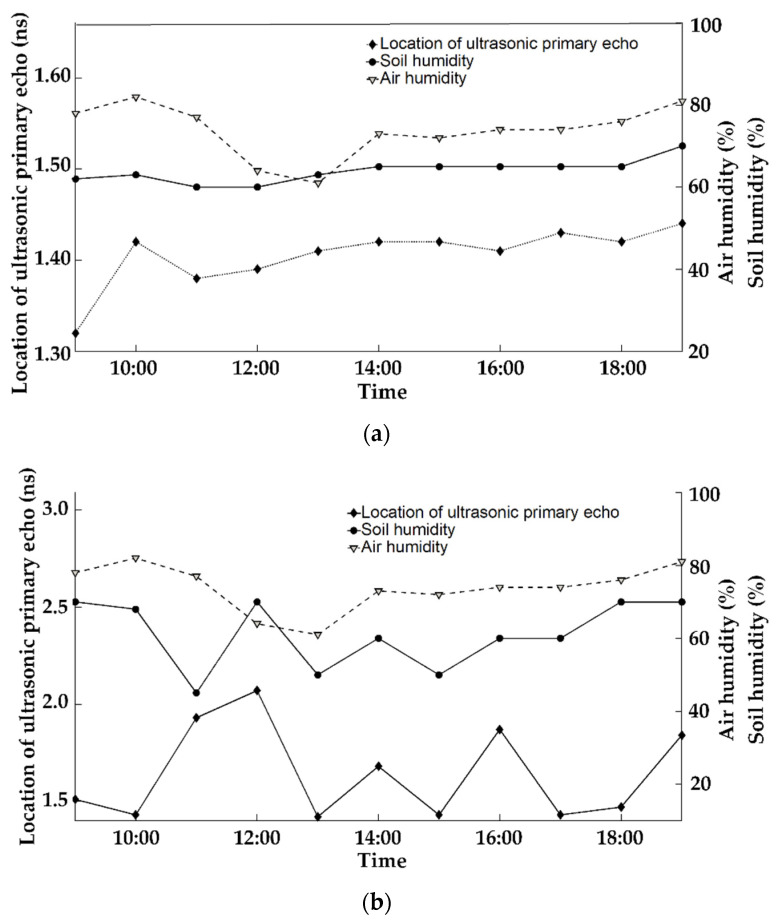
Changes of locations of ultrasonic echoes from the stem body of living sunflowers with the humidity of the soil and the air. (**a**) Sunflower sample 1; (**b**) sunflower sample 2.

Figure 7 shows two living sunflowers. From 12:00 to 18:00, the ultrasonic primary echo position and soil moisture have almost the same trends of changes. The average soil moisture value of sample 1 is 64%, the mean value of the ultrasonic primary echo position is 1.40 ns, and the average ultrasonic velocity is 1813 m/s. The average weight of the soil moisture of sample 2 is 61.8%, the mean value of primary ultrasonic echo is 1.65 ns, and the average ultrasonic velocity is 2844 m/s. The ultrasonic rate of the stem with water filling is lower than that of the stem with water shortage. This agrees with the experimental results of the above-water immersion test.

When the sunflower (sample 2) is short of water for a short time, at 12:00, the soil moisture is 70%, the ultrasonic echolocation is 2.07 ns, and the velocity is 2153 m/s. In order to maintain moisture, it shows that the sunflower restricts CO_2_ uptake and photosynthesis rate, and the transpiration activity is inhibited. Then, when soil moisture is adequate, the sunflower switch to normal water absorption and transpiration. At 13:00, transpiration activity was the most active all day; stem water decreased rapidly, and soil moisture decreased to 50%. The primary echo position fell to 1.42 ns, and the velocity speed is up to 3138 m/s.

When the sunflower (sample 1) is water-filling, from 11:00 to 14:00, the changes in the primary position are the most active. The velocity changes are moderated compared to the sunflower short of water.

## 4. Discussion and Conclusions

As non-homogeneous anisotropic non-metallic materials, there is a wide range of complex acoustic impedance interfaces within the stem, forming complex ultrasonic echo signals. The hybrid differential AIC algorithm can automatically detect the ultrasonic primary echo position of the plant stem. The experiment shows that using the hybrid difference AIC algorithm can achieve the accurate primary echo positions of stems, which lays the foundation for the ultrasonic detection of the plant stem.

The experiment of measured wood immersion shows that the primary echo position of the ultrasonic wave can effectively track the change in the water content of stems. However, when the ultrasonic attenuation of plant stems is too large, or the plant stems are too thick, the echo signal cannot be obtained even by increasing the ultrasonic emission energy, the method cannot be used.

The dynamic detection of the stem of a living sunflower can be carried out because of the non-destructive testing characteristics of ultrasound. The velocity of the stem when the sunflower is in the filling changes more actively than that in a water shortage. The results may present that the sunflower can regulate the water absorption and transpiration in balance in the filling condition, which ensures the stem has relatively constant water content. On the contrary, the sunflower would compress the absorption and transpiration in a water shortage. So, keeping the relatively regular water content in the stem is hard. 

It is assumed that sunflowers have apparent growth regulation for sunflower detection only in the daytime. Therefore, readings were only taken in the daytime, and we did not explore the differences between daytime and nighttime. 

Subsequent research can effectively intercept the primary echo signal after obtaining the primary echo’s location and analyze the dynamic growth’s spectrum characteristics and the change in the stem from the perspective of frequency energy.

To further analyze the characteristics of dynamic physiological changes of plant stems detected by ultrasound, we can design a contrastive analysis between ultrasonic detection and stem structure anatomy to explore features of dynamic physiological changes of the stems with the ultrasonic method.

## Figures and Tables

**Table 1:** Results of simulation signals.

Simulated Signals	The Minimal of AIC (ns)	Primary Echo Position (ns)	
		Observed	Hybrid
1	101	153	153
2	101	153	154
3	101	156	159
4	201	253	254
5	201	253	254
6	201	255	258
7	301	351	351
8	301	351	351
9	301	354	359

**Table 2:** Measured ultrasonic velocity, quality water content and volume water content of stems with different soaking times.

Parameter	Diameter/cm	The Time for Samples Immersed in Water														
		0	10 min	20 min	30 min	40 min	1 h	4 h	5 h	22 h	24 h	30 h	32 h	48 h	7D	9D
Quality/g	6.00	102.60	106.60	115.80	117.20	120.90	125.90	132.00	133.70	144.40	145.00	148.70	150.10	154.50	169.80	173.60
	7.00	111.20	122.60	125.70	127.30	129.70	132.90	141.70	144.00	157.00	160.00	165.40	166.00	172.00	191.50	196.70
	10.00	294.00	311.00	320.80	328.70	331.40	339.20	366.20	373.10	410.20	415.00	426.10	429.20	448.20	494.10	506.70
Density/(g·m^−3^)	6.00	0.60	0.63	0.68	0.69	0.71	0.74	0.78	0.79	0.85	0.85	0.88	0.88	0.91	1.00	1.02
	7.00	0.41	0.46	0.47	0.47	0.48	0.49	0.53	0.53	0.58	0.59	0.61	0.62	0.64	0.71	0.73
	10.00	0.37	0.40	0.41	0.42	0.42	0.43	0.47	0.48	0.52	0.53	0.54	0.55	0.57	0.63	0.65
Velocity/(m·s^−3^)	6.00	4.71 × 10^3^	4.35 × 10^3^	3.86 × 10^3^	3.48 × 10^3^	3.20 × 10^3^	3.04 × 10^3^	2.78 × 10^3^	2.76 × 10^3^	2.38 × 10^3^	2.34 × 10^3^	2.28 × 10^3^	1.95 × 10^3^	1.82 × 10^3^	1.63 × 10^3^	1.50 × 10^3^
	7.00	4.95 × 10^3^	4.42 × 10^3^	3.95 × 10^3^	3.69 × 10^3^	3.66 × 10^3^	3.50 × 10^3^	2.90 × 10^3^	2.88 × 10^3^	2.28 × 10^3^	2.11 × 10^3^	2.33 × 10^3^	2.03 × 10^3^	1.70 × 10^3^	1.56 × 10^3^	1.52 × 10^3^
	10.00	5.14 × 10^3^	4.55 × 10^3^	4.43 × 10^3^	4.34 × 10^3^	4.12 × 10^3^	3.97 × 10^3^	3.77 × 10^3^	3.68 × 10^3^	2.84 × 10^3^	2.77 × 10^3^	2.60 × 10^3^	2.49 × 10^3^	2.16 × 10^3^	1.74 × 10^3^	1.64 × 10^3^
Mass moisture content/%	6.00	0.00	0.04	0.11	0.12	0.15	0.19	0.22	0.23	0.29	0.29	0.31	0.32	0.34	0.40	0.41
	7.00	0.00	0.09	0.12	0.13	0.14	0.16	0.22	0.23	0.29	0.31	0.33	0.33	0.35	0.42	0.43
	10.00	0.00	0.05	0.08	0.11	0.11	0.13	0.20	0.21	0.28	0.29	0.31	0.32	0.34	0.40	0.42
volumetric moisture content/%	6.00	0.00	0.02	0.08	0.09	0.11	0.14	0.17	0.18	0.25	0.25	0.27	0.28	0.31	0.40	0.42
	7.00	0.00	0.04	0.05	0.06	0.07	0.08	0.11	0.12	0.17	0.18	0.20	0.20	0.23	0.30	0.32
	10.00	0.00	0.02	0.03	0.04	0.05	0.06	0.09	0.10	0.15	0.15	0.17	0.17	0.20	0.25	0.27

## Data Availability

Not applicable.
